# Photoinduced radical tandem annulation of 1,7-diynes: an approach for divergent assembly of functionalized quinolin-2(1H)-ones

**DOI:** 10.3389/fchem.2024.1371978

**Published:** 2024-03-26

**Authors:** Daixiang Chen, Zhi-Jie Song, Shenghu Yan, Guigen Li, Jia-Yin Wang, Yue Zhang

**Affiliations:** ^1^ School of Pharmacy, Changzhou University, Changzhou, Jiangsu, China; ^2^ Department of Chemistry and Biochemistry, Texas Tech University, Lubbock, TX, United States

**Keywords:** 1,7-diynes, photoinduced, Kharasch addition, annulative reactions, quinolin-2(1H)-ones

## Abstract

The first photocatalytic trichloromethyl radical-triggered annulative reactions of amide-linked 1,7-diynes with polyhalomethanes were established for the flexible assembly of functionalized quinolin-2(1H)-ones with generally acceptable yields. With the installation of the aryl group (R^1^) into the alkynyl moiety, *C*-center radical-initiated Kharasch-type addition/nucleophilic substitution/elimination cascade to produce quinolin-2(1H)-ones-incorporating *gem*-dihaloalkene, whereas three examples of polyhalogenated quinolin-2(1H)-ones were afforded when amide-linked 1,7-diynes bearing two terminal alkyne units were subjected to BrCX_3_ by exploiting dry acetonitrile as a solvent.

## Introduction

**FIGURE 1:**
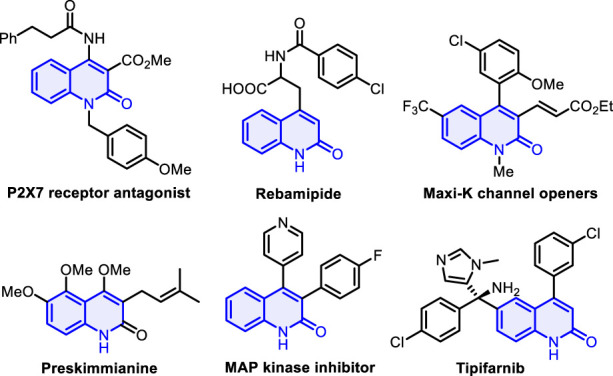
Selected examples of natural products and bioactive molecules containing quinolin-2(1H)-ones.

**SCHEME 1:**
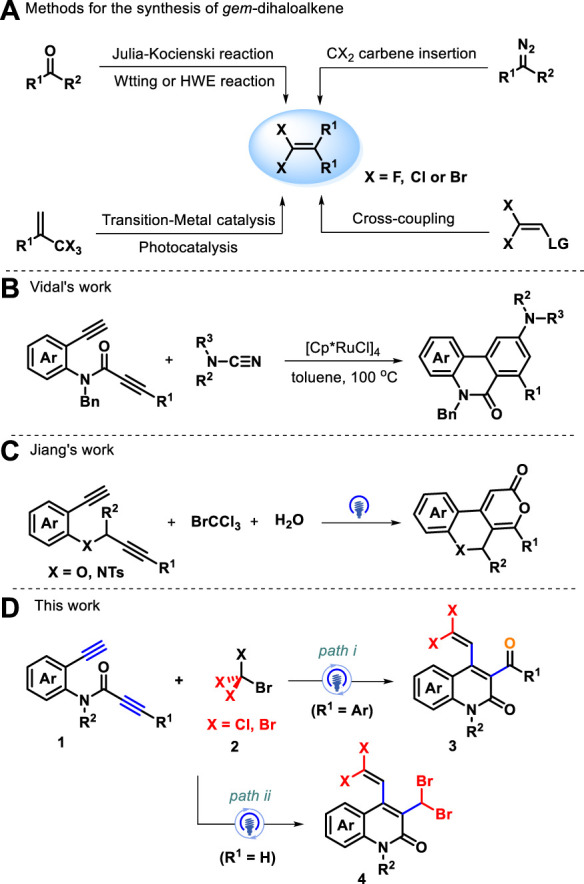
Methods for synthesizing gem-dihaloalkenes **(A)** and tandem annulation of 1,7-diynes **(B-C)**.

*Aza*-heterocyclic compounds are found in a wide variety of natural drugs and biologically active molecules, many of which are pharmacologically important (Pozharskii et al., 1997; Wen et al., 2022; Zhao et al., 2023; Liu et al., 2023a; Liu et al., 2023b). Among these, quinolin-2(1H)-one and its analogs are an important class of nitrogen-containing heterocycle scaffolds and are widely encountered in a myriad of pharmaceutical molecules and synthetic compounds (Sliskovic et al., 1991; Suzuki et al., 2001; Bach et al., 2002; Kuethe et al., 2005) which display versatile biological and pharmacological activities (McQuaid et al., 1992; Michael, 1995; Peifer et al., 2008), such as P2X7 receptor antagonist, rebamipide, and MAP kinase inhibitor (Figure 1) (Maignan et al., 2016; Tan et al., 2016; Miliutina et al., 2017; Wu et al., 2020). Various synthetic strategies have been achieved to construct the skeleton of such heterocycles, including Knorr synthesis (Liu et al., 2012; Ma et al., 2023), Friedlander reactions (Han et al., 2012), radical cyclization of acyclic precursors (Kadnikov and Larock, 2004; Manley and Bilodeau, 2004), and other methods (Fujita et al., 2004; Tsuritani et al., 2009; Berrino et al., 2012; Mai et al., 2014). The investigation of straightforward, atom-economic, environmentally acceptable, and green synthetic approaches to the construction of highly functionalized quinolin-2(1H)-ones remains a long-standing target and an active field of research in synthetic and medicinal chemistry. On the other hand, *gem*-dihaloalkenes are a unique structural unit with fascinating applications that range from organic synthesis to materials science (Rogawski, 2006; Meanwell, 2011) and can act as interesting synthetic intermediates in various chemical transformations for producing other useful molecules (Leriche et al., 2003; Okutami and Mori, 2009). Traditional approaches for the preparation of *gem*-dihaloalkenes include Wittig-type reactions, Julia–Kocienski reaction (Zhao et al., 2010; Chelucci, 2012; Zheng et al., 2013; Gao et al., 2015), and carbene insertion (Zeng et al., 2021) (Scheme 1A). With two geminal halogen atoms linked by an alkenyl carbon, these compounds exhibit higher reactivity for the oxidative addition of transition metal complexes than the corresponding monohaloolefins (London et al., 2014; Tian et al., 2016; Daniel et al., 2019), and the halogen atoms can be replaced by nucleophilic reagents through the additional elimination pathway (Yokota et al., 2007; Ichikawa et al., 2008). Despite significant progress in this field, the development of a new strategy for synthesizing a variety of valuable gem-dihaloalkenes remains a pressing need. To the best of our knowledge, the design and assembly of products incorporating a *gem*-dihaloalkene moiety and a quinolin-2(1H)-one skeleton using diynes as starting materials have not yet been reported.

Over the years, the tandem annulation of 1,*n*-diynes has become an applicable and attractive tool for the collection of isocyclic and heterocyclic compounds via synergistic processes across its carbon–carbon triple-bond *π* system in an atom-economical manner (Singidi et al., 2010; Wang et al., 2017; Chintawar et al., 2019). For instance, Vidal and colleagues established Ru-catalyzed [2+2+2] cycloaddition of amide-linked 1,7-diynes with electron-rich cyanamide for forming benzo[*c*][2,7]naphthyridinones as a major product in good yields and regioselectivities (Scheme 1B) (Huvelle et al., 2022). Additionally, photocatalytic Kharasch-type-addition cyclization of 1,*n*-diynes provides another sustainable way of yielding various functionalized ring structures (Wang et al., 2021; Wu et al., 2021; Zheng et al., 2021). Recently, Jiang’s group elaborated a photocatalytic three-component biheterocyclization of heteroatom-linked 1,7-diynes with CBrCl_3_ and water as oxygen sources, leading to access of skeletally diverse fused-tricyclic heterocycles (Scheme 1C) (Wang et al., 2021). Intrigued by previous work and the continuation of our interest in radical cascade reactions (Wang et al., 2023a; Wang et al., 2023b; Wang et al., 2023c; Zhang et al., 2023), we believed that CCl_3_ radical derived from BrCCl_3_ under visible-light irradiation could add to preformed amide-linked 1,7-diynes followed by 6-*exo*-*dig* cyclization, 1,5-(S_N_″)-substitution, and dehydrohalogenation to furnish versatile functionalized quinolin-2(1H)-ones. No construction of quinolin-2(1H)-ones bearing *gem*-dihaloalkenes starting from 1,7-diynes and perhalogenated methanes has been reported. As anticipated, photocatalytic radical-induced addition-annulation was enabled by the reaction of amide-tethered 1,7-diynes **1** with bromotrichloromethane **2** in the presence of NaHCO_3_ to provide densely decorated 3-benzoyl-4-(2,2-dichlorovinyl)quinolin-2(1H)-ones **3** (Scheme 1D, *path i*). Notably, this reaction could obtain 3-(dibromomethyl)-4-(2,2-dichlorovinyl)quinolin-2(1H)-ones **4** when two terminal alkynes were installed into amide-tethered 1,7-diynes (Scheme 1D, *path ii*). We thus report these two types of interesting transformations.

## Results and discussion

Initially, *N*-benzyl-*N*-(2-ethynylphenyl)-3-phenylpropiolamide **1a** and CBrCl_3_ **2a** were selected as representative substrates under the irradiation of 30 W blue LEDs to identify the reaction conditions (Table 1). With eosin Y or Mes-Acr^+^ClO_4_^−^ as photocatalysts, the reaction in the presence of K_2_CO_3_ in acetonitrile at room temperature did not detect the desired product **3a** (entries 1–2). Fortunately, the use of *fac*-Ir(ppy)_3_ as a photocatalyst could drive the conversion of **1a** into **3a**, although the yield of quinolin-2(1H)-one **3a** was 28% (entry 3). Next, we screened other inorganic and organic bases, comprising Na_2_CO_3_, KOAc, Na_3_PO_4_, NaHCO_3_, Na_2_HPO_4_, 4-dimethylaminopyridine (DMAP), and Et_3_N, for this photocatalysis by using *fac*-Ir(ppy)_3_ as the photocatalyst (entries 4–10). After careful screening, NaHCO_3_ was determined as the best choice, providing **3a** at a higher 62% yield (entry 15). Based on *fac*-Ir(ppy)_3_ as a photocatalyst and NaHCO_3_ as a base, we then tested the solvent effect by screening several other solvents such as 1,2-dichloroethane (DCE, 33%), toluene (25%), 1,4-dioxane (22%), tetrahydrofuran (THF, NR), and EtOH (32%). The use of THF completely suppressed the reaction process, whereas other solvents we attempted gave more reduced yields than MeCN (entries 11–15).

**TABLE 1:** Optimization conditions for forming **3a** ^a^.

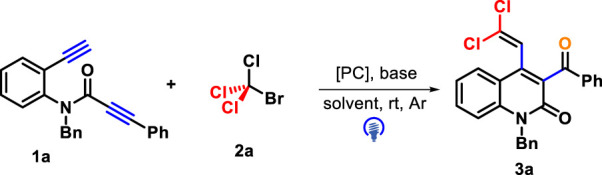				
Entry	PC	Base	Solvent	Yield (%)^b^
1	Eosin Y	K_2_CO_3_	CH_3_CN	ND
2	Mes^−^Acr^+^ClO_4_^−^	K_2_CO_3_	CH_3_CN	ND
3	*fac*-Ir(ppy)_3_	K2CO3	CH_3_CN	28
4	*fac*-Ir(ppy)_3_	Na_2_CO_3_	CH_3_CN	30
5	*fac*-Ir(ppy)_3_	KOAc	CH_3_CN	41
6	*fac*-Ir(ppy)_3_	Na_3_PO_4_	CH_3_CN	37
7	*fac*-Ir(ppy)_3_	NaHCO_3_	CH_3_CN	62
8	*fac*-Ir(ppy)_3_	Na_2_HPO_4_	CH_3_CN	11
9	*fac*-Ir(ppy)_3_	Et_3_N	CH_3_CN	Trace
10	*fac*-Ir(ppy)_3_	DMAP	CH_3_CN	Trace
11	*fac*-Ir(ppy)_3_	NaHCO_3_	DCE	33
12	*fac*-Ir(ppy)_3_	NaHCO_3_	Toluene	25
13	*fac*-Ir(ppy)_3_	NaHCO_3_	1,4-Dioxane	22
14	*fac*-Ir(ppy)_3_	NaHCO_3_	THF	NR
15	*fac*-Ir(ppy)_3_	NaHCO_3_	EtOH	32

^a^ All reaction conditions were performed in 1,7-diyne **1a** (0.1 mmol), BrCCl_3_ (0.2 mmol), [PC] (1.0 mol%), base (2.0 equiv), solvent (1.0 mL) under 30 W blue LEDs, irradiation, at room temperature, under Ar atmosphere for 12 h.

^b^ Isolated yield based on **1a**. ND, not detected; NR, no reaction.

**SCHEME 2:**
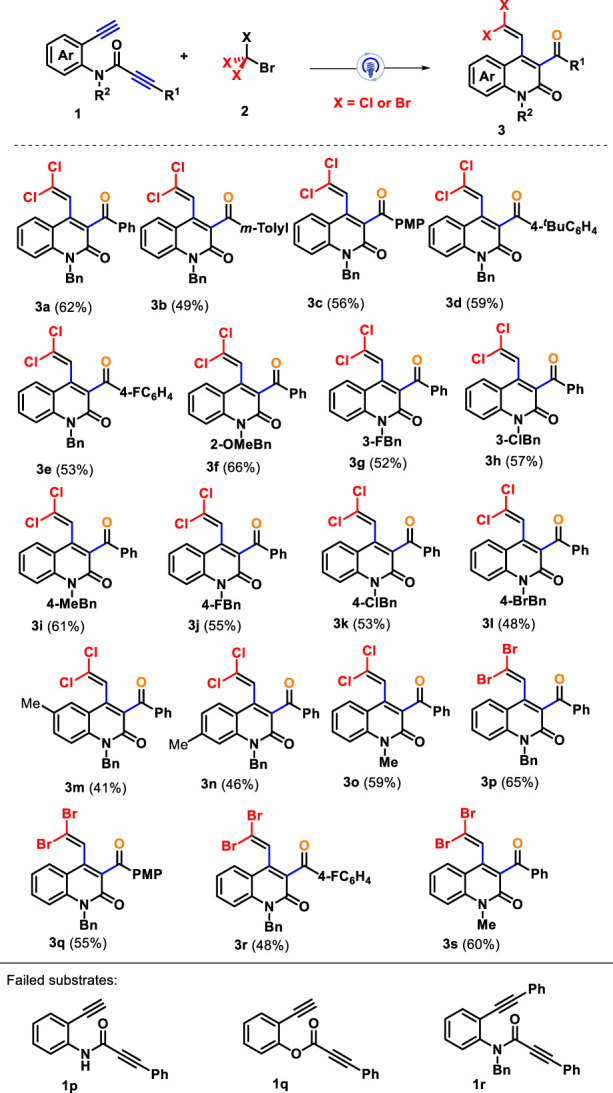
Substrate scope for synthesizing product **3**

Having establishing the optimal reaction conditions, we then evaluated the substrate scope and generality of an array of amide-linked 1,7-diynes for this photocatalytic radical tandem annulation toward synthesizing quinolin-2(1H)-ones bearing *gem*-dihaloalkenes; the results are summarized in Scheme 2. First, CBrCl_3_ (**2a**) reacted with 1,7-diynes **1** to investigate the influence of different the electronic properties and positions of substituents in the arylalkynyl units (R^1^), and all of them conveniently participated in the current cascade cyclization with acceptable yields. Both electron-donating (such as methyl **1b**, methoxy **1c**, and *tert*-butyl **1d**) and electron-withdrawing (fluoro **1e**) groups located at the *para*- or *meta-*position of the arylalkynyl moiety all performed well in this transformation, affording the corresponding *gem*-dichloroalkenes **3b**–**3e** in 49%–59% yields. However, the obvious impact on steric hindrance and electronic effect was demonstrated because arylalkynyl with *ortho*-substituted or strong electron-withdrawing groups were suppressed during the reaction process, delivering almost no desired product. Subsequently, 1,7-diynes with different benzyl groups of nitrogen atoms could perform smoothly under standard conditions. The benzyl group bearing a functional group, including ether (*o*-methoxy **1f**), alkyl (*p*-methyl **1i**), and halogen (*m*-fluoro **1g**, *m*-chloro **1h**, *p*-fluoro **1j**, *p*-chloro **1k**, and *p*-bromo **1l**), proved to be good candidates for the reaction, enabling their addition-cyclization to render the desired products **3f**–**3l** with yields ranging from 48% to 66%. Subsequently, we chose methyl (**1m** and **1n**) as the representative functional group to introduce the C4 or C5 position of the internal arene ring of 1,7-diynes to investigate its synthesis efficiency. The corresponding products **3m**–**3n** were isolated in 41% and 46% yields, respectively. Furthermore, for the replacement of the benzyl group with a methyl group on the nitrogen atoms, amide-tethered 1,7-diynes **1o** was a good reaction analog, giving the product **3o** with a yield of 59%. Similarly, the substrate scope of this method was further assessed by taking advantage of CBr_4_ as the *gem*-dibromination reagent for assembling *gem*-dibromovinyl-incorporating quinolin-2(1H)-ones. We found that 1,7-diynes **1** with varied substitution patterns could effectively take part in the current system, furnishing corresponding products **3p**–**3s** in 48%–65% yields. Unfortunately, N-unprotected amide-linked 1,7-diyne **1p** and ester-linked 1,7-diyne **1q** did not yield desired products. In addition, the preformed substrate **1r** with two internal alkyne moieties was an unreactive reactant under standard conditions, and 1,7-diyne **1r** was recovered, showing that terminal alkynes on starting material play an important role in this transformation.

**SCHEME 3:**
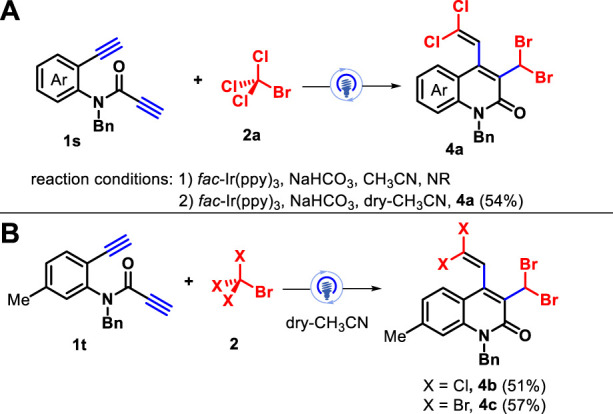
Synthesis of polyhalogenated quinolin-2(1H)-one 4a **(A)** and 4b-4c **(B)**.

To further expand the range of substrates for this transformation, amide-linked 1,7-diynes with two terminal alkynyl moieties **1s** were subjected to the reaction of CBrCl_3_ under the above optimal conditions, but the reaction was completely suppressed. Surprisingly, the reaction can proceed smoothly in the presence of dry acetonitrile, and the unprecedented polyhalogenated quinolin-2(1H)-ones **4a** was obtained in 54% yield *via* 1,5-(SN″)-substitution (Scheme 3A). Furthermore, a moderate chemical yield was observed for the 1,7-diynes with a methyl group located at the 5-position of the internal arene ring **1t** for the assembly of the polyhalogenated products **4b**–**4c** (Scheme 3B). The structures of densely functionalized quinolin-2(1H)-ones **3** and **4** were fully characterized by their NMR spectroscopy and HRMS date, and two cases of **3a** and **4a** were confirmed by X-ray diffraction analysis (see Supplementary Material).

**SCHEME 4:**
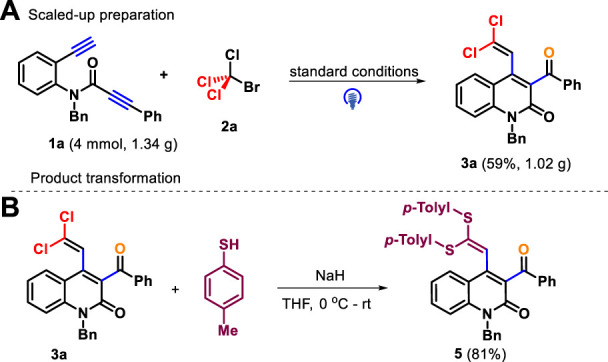
Scaled-up preparation **(A)** and product transformation **(B)**.

The gram-scale experiments for the preparation of **3a** on a 4.0 mmol scale were conducted under optimal conditions, and the product was delivered with a comparable yield (59%, Scheme 4A). The practicality of this methodology was further studied through the synthetic application of products. For example, the double nucleophilic vinylic substitution reaction **3a** and *p*-toluenethiol proceeded smoothly by means of sodium hydride as base, which led to the product **5** in 81% yield (Scheme 4B) (Jiang et al., 2017).

**SCHEME 5:**
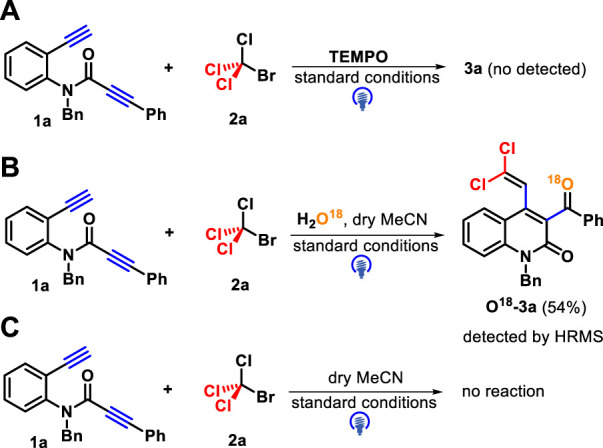
Mechanistic experiments **(A-C)**.

**FIGURE 2:**
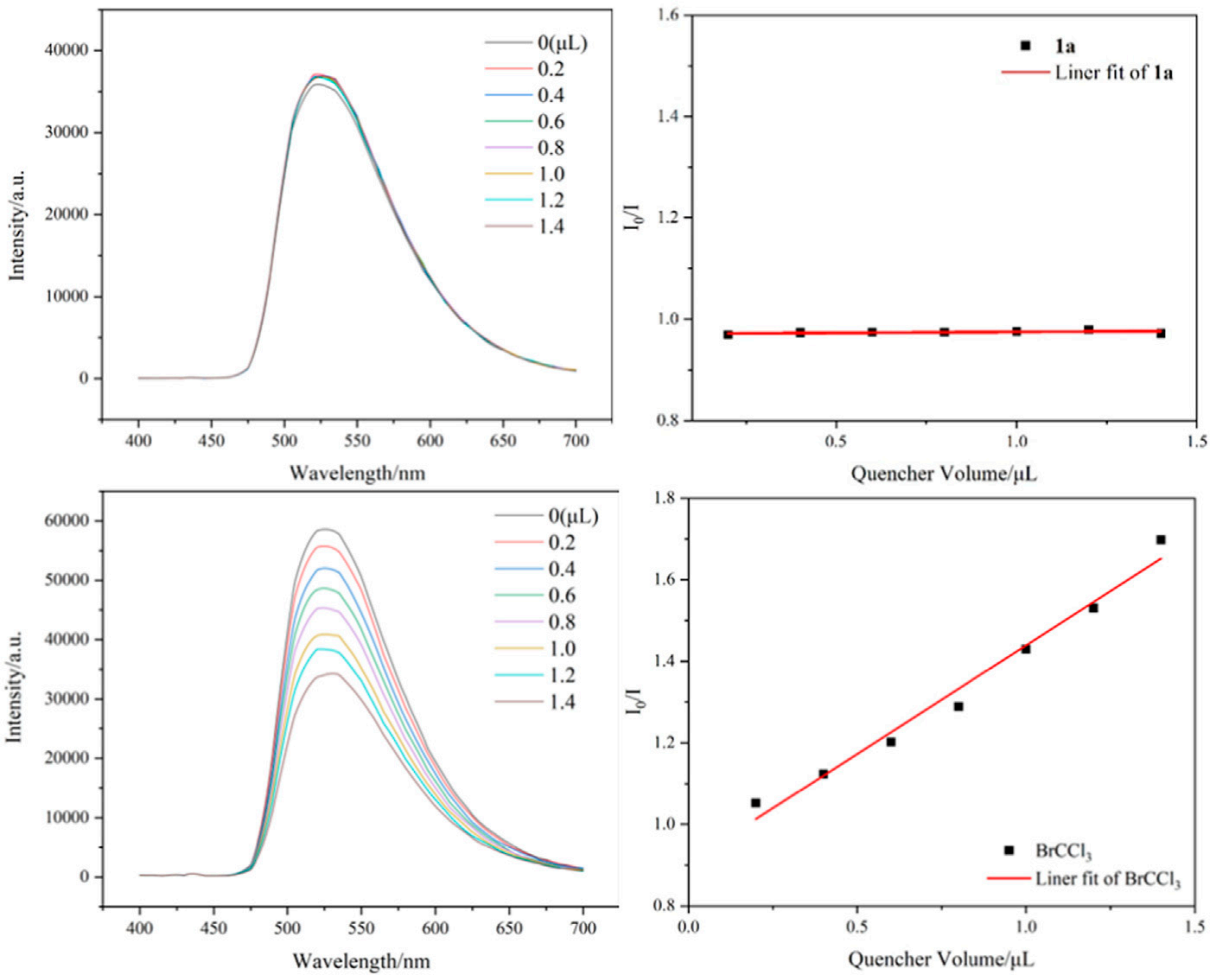
Stern–Volmer analysis for *fac*-Ir(ppy)_3_ with **1a** and BrCCl_3_ **2a**.

Several control experiments were performed to gain insights into the reaction pathway mechanism. First, the use of a radical inhibitor TEMPO (2,2,6,6-tetramethyl-1-piperidinyloxy) successfully suppressed the reaction process, and the result confirmed that a trichloromethyl radical may be involved in these transformations (Scheme 5A). Next, the reaction occurred in the presence of H_2_O^18^, and the product containing O^18^ was isolated in 54% yield and identified by HR-MS (Scheme 5B). In addition, when dry CH_3_CN was employed as a solvent under standard conditions, the reaction progress was completely inhibited (Scheme 5C). These two survey results showed that the oxygen source of the carbonyl group in target products comes from water. Finally, several fluorescence quenching experiments indicated that CBrCl_3_ **2a** was a more efficient quencher of the excited state of *fac*-Ir(ppy)_3_* than 1,7-diyne **1a** (Figure 2).

**SCHEME 6:**
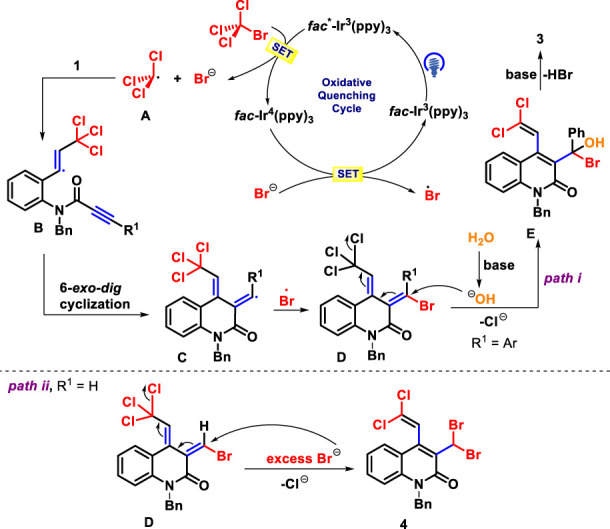
Plausible reaction pathway for forming **3** and **4**.

In light of these findings and previous related works (Wang et al., 2021; Wu et al., 2021; Zheng et al., 2021), we propose a plausible mechanism for this photo-catalyzed annulation of 1,7-diynes, as shown in Scheme 6. The photocatalytic cycle was initiated by the activation of Ir(III) with blue light irradiation to form the excited state Ir(III)* species, which reacts with BrCCl_3_ to yield trichloromethyl radical **A** and a bromine anion, together with Ir(IV) complex via a single electron transfer (SET). Next, the radical **A** can be trapped by the terminal carbon-carbon triple bond of 1,7-diyne **1** to provide the alkenyl radical **B**, which undergoes 6-*exo*-*dig* cyclization to give intermediate **C**. The resulting bromine anion was oxidized by Ir(IV) complex to produce Br radical (Bacauanu et al., 2018; Wang et al., 2019), followed by radical cross coupling with **C** to obtain intermediate **D** and regenerate Ir(III) species. Subsequently, the intermediate **D** reacts with OH^−^ from H_2_O to afford the intermediate **E** through 1,5-(S_N_″)-substitution, which eliminates one molecule of HBr to assemble the desired product **3** (*path i*). The latter process, different from the above, undergoes 1,5-(S_N_″)-nucleophilic substitution with excess Br^−^ in the photocatalytic system to give polyhalogenated products **4** (*path ii*).

## Conclusion

Starting from new prepared amide-anchored 1,7-diynes, and easily available polyhalomethanes, we have illustrated a practical photocatalytic 6-*exo*-*dig* cyclization of 1,7-diynes, enabling substrate-controlled divergent synthesis of two types of functionalized quinolin-2(1H)-ones in moderate to excellent yields. When the aryl group (R^1^) was introduced into the alkynyl unit of 1,7-diynes, photoinduced radical cyclization cascades to access *gem*-dihaloalkene-containing quinolin-2(1H)-ones. Significantly, 1,7-diynes bearing two terminal alkynes were employed to react with BrCX_3_ by using dry acetonitrile as solvents, unexpectedly delivering three examples of polyhalogenated quinolin-2(1H)-ones. This reaction system features bond-forming efficiency, broad functional group compatibility, and mild reaction conditions. Further research on this amide-linked 1,7-diyne is currently being conducted by our group.

## Data Availability

The original contributions presented in the study are included in the article/Supplementary Material; further inquiries can be directed to the corresponding authors.
