# Supplementary material for: Photoinduced radical tandem annulation of 1,7-diynes: an approach for divergent assembly of functionalized quinolin-2(1H)-ones
Source: Front Chem. 2024 Mar 26;12:1371978. doi: 10.3389/fchem.2024.1371978 (PMC11002210; doi:10.3389/fchem.2024.1371978)
Supplement: Supplementary file 1 [file DataSheet1.docx]

Supporting Information

**Photoinduced Radical Tandem Annulation of 1,7-Diynes: An Approach for Divergent Assembly of Functionalized Quinolin-2(1H)-ones**

Daixiang Chen,^a^ Zhi-Jie Song,^a^ Shenghu Yan,^a^ Guigen Li,^b,^ Jia-Yin Wang,*^a^ Yue Zhang*^a^

^a^School of Pharmacy, Changzhou University, Changzhou, Jiangsu 213164, China; email: wjychem@cczu.edu.cn (W.JY.); [zyjs@cczu.edu.cn](mailto:zyjs@cczu.edu.cn) (Z.Y.)

^b^Department of Chemistry and Biochemistry, Texas Tech University, Lubbock 79409-1061, Texas, USA; email: guigen.li@ttu.edu (G.L.)

| General Information…………………………………………………………………….. | S2 |
| --- | --- |
| Preparation of Substrates **1**…………………………….…………………………..… | S2 |
| Luminescence Quenching Experiment………………………………………………… | S2-S3 |
| Control Experiment with H_2_^18^O……………………………………………………… | S3 |
| X-ray Crystallography Structure……………………………………………………… | S3-S4 |
| General Procedure for the Synthesis of Compound **5**…………………………………. | S4 |
| Characterization Data of Compound **5**……………………………………………..… | S4 |
| General Procedure for the Synthesis of Compound **3** and **4**……………………………. | S4-S5 |
| Characterization Data of Compound **3** and **4**………………………………………..… | S5-S11 |
| Copies of ^1^H and ^13^C NMR Spectra for Compounds **3**, **4** and **5**…………………….… | S12-S57 |

**General Information**

^1^H NMR (^13^C NMR) spectra were measured on a Bruker DPX 400 MHz spectrometer in CDCl_3_ (DMSO-*d_6_*) with chemical shift (*δ*) given in ppm relative to TMS as internal standard [(s = singlet, d = doublet, t = triplet, brs = broad singlet, m = multiplet), coupling constant (Hz)]. HRMS (ESI) was determined by using microTOF-QII HRMS/MS instrument (BRUKER). The melting points were measured with digital melting point detector. PE refers to petroleum ether (bp 60-90 °C), and EA refers to ethyl acetate. All reagents (analytical pure) were purchased from commercial suppliers, Aladdin, Adamas-beta® and Energy Chemical unless otherwise noted and used without further purification. X-Ray crystallographic analysis was performed with a Siemens SMART CCD and a Siemens P4 diffractomete.

**Preparation of Substrates 1**

1,7-diynes are known compounds and were prepared according to literature procedures (*Org. Lett.* **2022**, *24*, 5126-5131; *Org. Lett*. **2018**, *20*, 6765-6768). The general procedure was described using substrate **1a** as example.

**Luminescence Quenching Experiment**

The luminescence quenching experiment was taken using a FS5 Spectrophotometer (Edinburgh FS5). The excitation wavelength was 387 nm. The emission intensity was collected at 529 nm. The samples were prepared by mixing *fac*-Ir(ppy)_3_ (1.0× 10^-4^ mol/L) and different amount of quenchers (1,7-diyne **1a** and BrCCl_3_) in MeCN (total volume = 2.0 mL) in a light path quartzfluorescence cuvette. The concentration of 1,7-diyne (**1a**) stock solution is 1.0× 10^-4^ mol/L in MeCN. The concentration of BrCCl_3_ stock solution is 1.0× 10^-3^ mol/L in MeCN. For each quenching experiment, each volume of quenchers stock solution was titrated to a mixed solution of *fac*-Ir(ppy)_3_ (20, 20, 20, 20, 20, 20, 20×10^-3^ mL, in a total volume = 2.0 mL). Then the emission intensity was collected and the results were presented in Figure S1 and Figure S2.


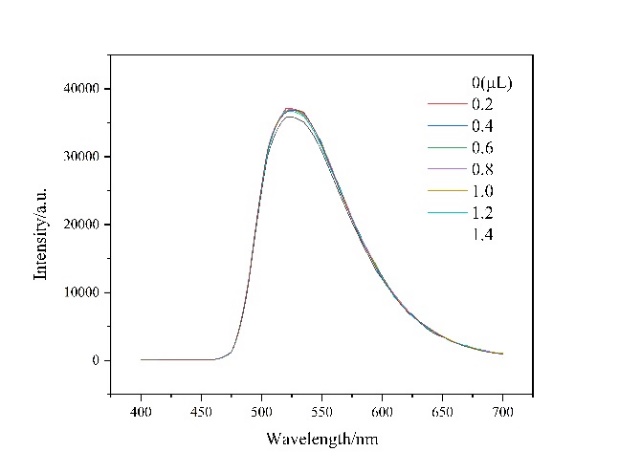

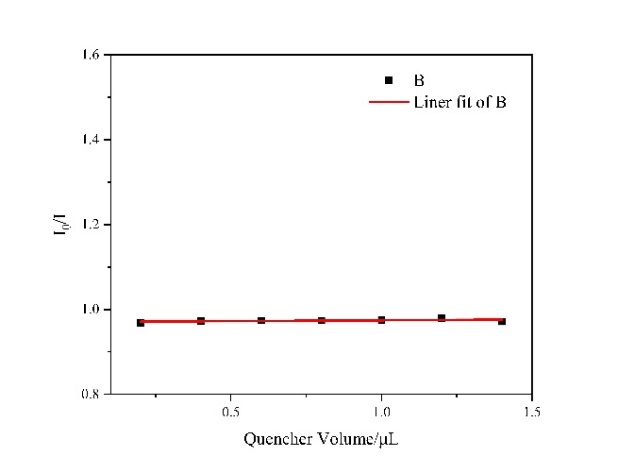


**Figure S1**. Stern−Volmer analysis for *fac*-Ir(ppy)_3_ with **1a**


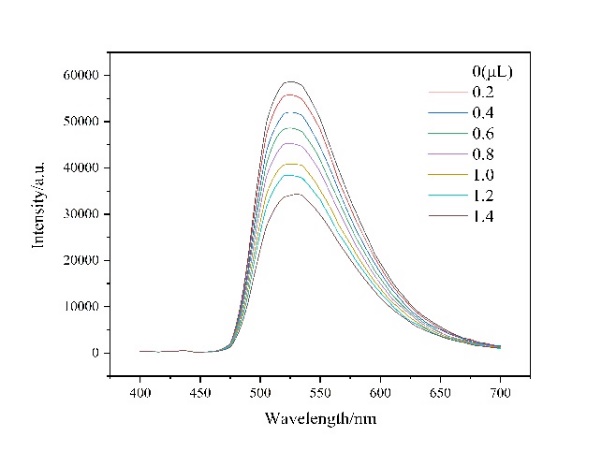
**
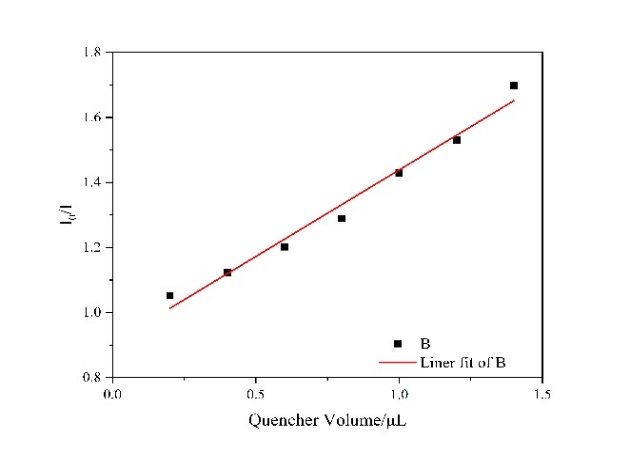
**

**Figure S2**. Stern−Volmer analysis for *fac*-Ir(ppy)_3_ with BrCCl_3_

**Control Experiment with H_2_^18^O**

In a 10-mL Schlenk tube, 1,7-diyne **1a** (0.1mmol, 33.5 mg, 1.0 equiv), BrCCl_3_ (0.2 mmol, 79.2 mg, 2.0 equiv), NaHCO_3_ (0.2 mmol, 16.8 mg, 2.0 equiv), *fac*-Ir(ppy)_3_ (0.6 mg, 1 mol%), H_2_O^18^ (0.2 mmol, 4 mg, 2 equiv) and dry-MeCN (1.0 mL) were successively added under Ar conditions. Then, the tube was stirred at room temperature for 12 h under 30 W blue light irradiation until complete consumption of **1a** as monitored by TLC analysis. After the reaction was completed, O^18^-containing product **3a** was detected by HR-MS.

**Figure S3. Copy of HR-MS Spectrum of O^18^-containing Product 3a**

**X-ray Crystallography Structure**


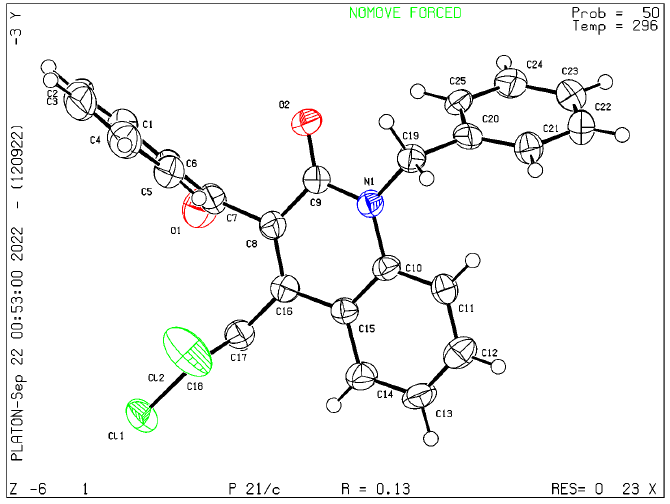


**Figure S4**. X-Ray Structure of Product **3a** (the ellipsoid contour 30% probability levels)

**CCDC** (2294736)


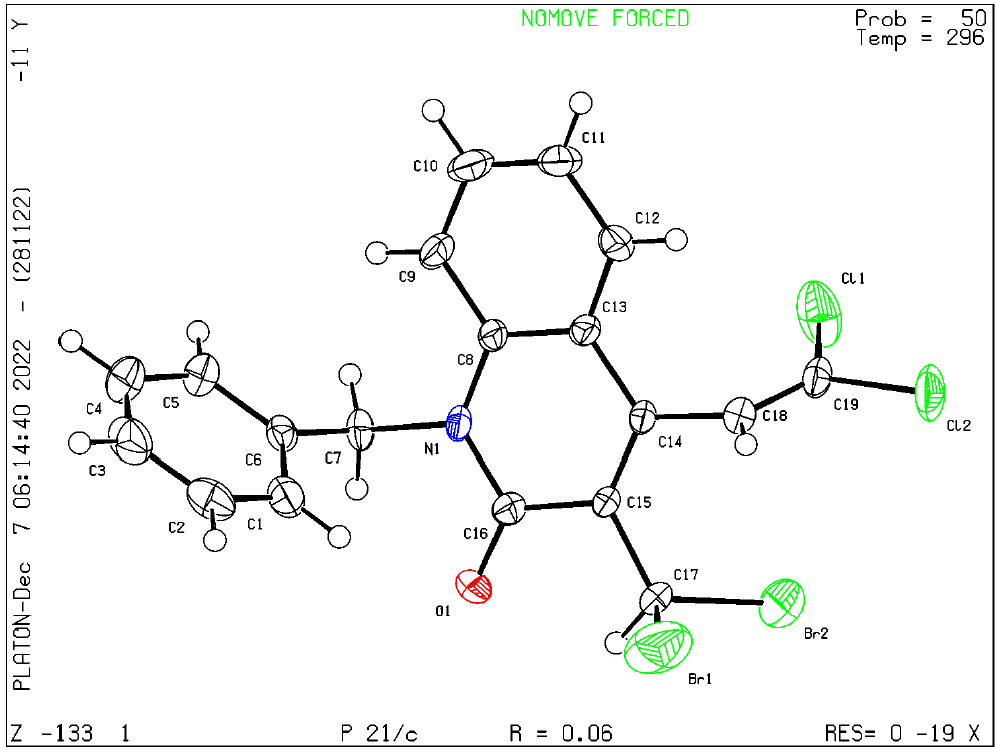


**Figure S4**. X-Ray Structure of Product **4a** (the ellipsoid contour 30% probability levels)

**CCDC** (2294785)

**General procedure for the synthesis of compounds 5**

NaH (7.2 mg, 0.3 mmol, 3.0 equiv) was added to **3a** (43.3 mg, 0.1 mmol, 1.0 equiv) in dry THF (2 mL) and stirred for 1 hour at 0 ^o^C. Then *p*-toluene mercaptan (37.2 mg, 0.3 mmol, 3.0 equiv) was added, move the mixture to room temperature for overnight. The mixture was then quenched with saturated NH_4_Cl and extracted with EtOAc. The organic phase was concentrated and evaporated on a rotary evaporator. The crude product was purified by silica gel chromatography to obtain the compound **5** as white solid.

***3-benzoyl-1-benzyl-4-(2,2-bis(p-tolylthio)vinyl)quinolin-2(1H)-one (5)***

White solid after purification by column chromatography (petroleum ether/ethyl acetate = 10/1); 49.3 mg, 81% yield; mp: 154-156 ^o^C;^1^H NMR (400 MHz, CDCl_3_) (δ, ppm): 8.15-8.10 (m, 2H), 7.72 (d, *J* = 12.4 Hz, 1H), 7.38-7.28 (m, 6H), 7.27-7.20 (m, 3H), 7.19-7.15 (m, 4H), 7.07-7.03 (m, 2H), 6.96 (d, *J* = 8.0 Hz, 4H), 5.91 (s, 1H), 5.43 (s, 2H), 2.17 (s, 6H). HRMS (ESI) m/z calcd for C_39_H_31_NNaO_2_S_2_ [M+Na]^+^ 632.1694, found 632.1689.

**General procedure for the synthesis of compounds 3 and 4**

Example for the synthesis of **3a**:

In a 10-mL Schlenk tube, 1,7-diyne **1a** (0.2 mmol, 67.0 mg, 1.0 equiv.), BrCCl_3_ (0.4 mmol, 79.2 mg, 2.0 equiv.), NaHCO_3_ (0.4 mmol, 33.6 mg, 2.0 equiv), *fac*-Ir(ppy)_3_ (1.2 mg, 1 mol%) and MeCN (2.0 mL) were successively added under Ar conditions. Then, the tube was stirred at room temperature for 12 h under 30 W blue light irradiation until complete consumption of **1a** as monitored by TLC analysis. After the reaction was completed, the reaction mixture was concentrated in vacuum and the resulting residue was purified by column chromatography on silica gel (eluent, petroleum ether/ethyl acetate = 15:1) to afford the desired product **3a** (53.7 mg, 62%) as a white solid.

Scaled-up preparation product **3a**:

In a 50-mL Schlenk tube, 1,7-diyne **1a** (4 mmol, 1.34 g, 1.0 equiv.), BrCCl_3_ (8 mmol, 1.58 g, 2.0 equiv.), NaHCO_3_ (8 mmol, 0.67 g, 2.0 equiv), *fac*-Ir(ppy)_3_ (26.2 mg, 1 mol%) and MeCN (30.0 mL) were successively added under Ar conditions. Then, the tube was stirred at room temperature for 15 h under 30 W blue light irradiation until complete consumption of **1a** as monitored by TLC analysis. After the reaction was completed, the reaction mixture was concentrated in vacuum and the resulting residue was purified by column chromatography on silica gel (eluent, petroleum ether/ethyl acetate = 15:1) to afford the desired product **3a** (1.02 g, 59%) as a white solid.

***3-benzoyl-1-benzyl-4-(2,2-dichlorovinyl)quinolin-2(1H)-one (3a)***

White solid after purification by column chromatography (petroleum ether/ethyl acetate = 15/1); 53.7 mg, 62% yield; mp: 133-135 ^o^C; ^1^H NMR (400 MHz, CDCl_3_) (δ, ppm): 8.02-7.91 (m, 2H), 7.75-7.73 (m, 1H), 7.65-7.41 (m, 5H), 7.37-7.27 (m, 6H), 6.88 (s, 1H), 5.60 (s, 2H). ^13^C NMR (100 MHz, CDCl_3_) (δ, ppm): 193.6, 159.8, 141.4, 139.6, 136.4, 135.9, 133.8, 132.0, 131.3, 129.3, 128.9, 128.7, 127.5, 127.4, 127.3, 126.9, 122.9, 122.4, 118.3, 115.6, 46.2. HRMS (ESI) m/z calcd for C_25_H_17_^35^Cl_2_NO_2_Na [M+Na]^+^ 456.0534, found 456.0528; C_25_H_17_^37^Cl_2_NO_2_Na [M+Na]^+^ 458.0505, found 458.0504.

***1-benzyl-4-(2,2-dichlorovinyl)-3-(3-methylbenzoyl)quinolin-2(1H)-one (3b)***

White solid after purification by column chromatography (petroleum ether/ethyl acetate = 15/1); 43.8 mg, 49% yield; mp: 138-140 ^o^C; ^1^H NMR (400 MHz, CDCl_3_) (δ, ppm): 7.76-7.70 (m, 3H), 7.57-7.53 (m, 1H), 7.42 (d, *J* = 8.8 Hz, 2H), 7.38-7.27 (m, 7H), 6.85 (s, 1H), 5.57 (s, 2H), 2.39 (s, 3H). ^13^C NMR (100 MHz, CDCl_3_) (δ, ppm): 193.7, 159.7, 141.2, 137.6, 136.5, 136.0, 133.7, 133.3, 132.6, 131.2, 129.4, 129.3, 128.9, 128.6, 127.5, 127.0, 126.9, 126.9, 122.6, 118.3, 115.5, 46.1, 20.8. HRMS (ESI) m/z calcd for C_26_H_19_^35^Cl_2_NO_2_Na [M+Na]^+^ 470.0691, found 470.0682; C_26_H_19_^37^Cl_2_NO_2_Na [M+Na]^+^ 472.0661, found 472.0660;

***1-benzyl-4-(2,2-dichlorovinyl)-3-(4-methoxybenzoyl)quinolin-2(1H)-one (3c)***

White solid after purification by column chromatography (petroleum ether/ethyl acetate = 15/1); 51.9 mg, 56% yield; mp: 137-139 ^o^C; ^1^H NMR (400 MHz, CDCl_3_) (δ, ppm): 7.92 (d, *J* = 8.8 Hz, 2H), 7.72-7.70 (m, 1H), 7.57-7.52 (m, 1H), 7.40 (d, *J* = 8.8 Hz, 1H), 7.34-7.26 (m, 7H), 6.95 (d, *J* = 8.8 Hz, 2H), 6.85 (s, 1H), 5.57 (s, 2H), 3.88 (s, 3H). ^13^C NMR (100 MHz, CDCl_3_) (δ, ppm): 191.9, 164.2, 159.8, 140.9, 139.5, 136.0, 131.8, 131.6, 129.5, 128.9, 127.5, 127.3, 127.1, 126.9, 122.8, 122.5, 118.3, 115.6, 114.0, 55.5, 46.2. HRMS (ESI) m/z calcd for C_26_H_19_^35^Cl_2_NO_3_Na [M+Na]^+^ 486.0640, found 486.0632; C_26_H_19_^37^Cl_2_NO_3_Na [M+Na]^+^ 488.0610, found 488.0605.

***1-benzyl-3-(4-(tert-butyl)benzoyl)-4-(2,2-dichlorovinyl)quinolin-2(1H)-one (3d)***

White solid after purification by column chromatography (petroleum ether/ethyl acetate = 15/1); 57.7 mg, 59% yield; mp: 144-146 ^o^C; ^1^H NMR (400 MHz, CDCl_3_) (δ, ppm): 7.86 (d, *J* = 8.4 Hz, 2H), 7.73-7.71 (m, 1H), 7.56-7.52 (m, 1H), 7.49-7.46 (m, 2H), 7.41 (d, *J* = 8.4 Hz, 1H), 7.34-7.26 (m, 6H), 6.87 (s, 1H), 5.57 (s, 2H), 1.34 (s, 9H). ^13^C NMR (100 MHz, CDCl_3_) (δ, ppm): 193.1, 159.9, 157.6, 141.2, 139.6, 135.9, 133.8, 131.8, 131.6, 129.3, 128.9, 127.5, 127.3, 127.2, 126.9, 125.7, 122.9, 122.5, 118.3, 115.6, 46.2, 35.2, 31.1. HRMS (ESI) m/z calcd for C_29_H_25_^35^Cl_2_NO_2_Na [M+Na]^+^ 512.1160, found 512.1157; C_29_H_25_^37^Cl_2_NO_2_Na [M+Na]^+^ 514.1131, found 514.1134.

***1-benzyl-4-(2,2-dichlorovinyl)-3-(4-fluorobenzoyl)quinolin-2(1H)-one (3e)***

White solid after purification by column chromatography (petroleum ether/ethyl acetate = 15/1); 47.8 mg, 53% yield; mp: 129-131 ^o^C; ^1^H NMR (400 MHz, CDCl_3_) (δ, ppm): 7.99-7.93 (m, 2H), 7.73-7.71 (m, 1H), 7.58-7.54 (m, 1H), 7.42 (d, *J* = 8.4 Hz, 1H), 7.35-7.26 (m, 6H), 7.17-7.13 (m, 2H), 6.87 (s, 1H), 5.57 (s, 2H). ^13^C NMR (100 MHz, CDCl_3_) (δ, ppm): 192.0, 166.2 (d, *J* = 256.0 Hz), 159.8, 141.6, 139.6, 135.8, 132.9 (d, *J* = 2.9 Hz), 132.1, 132.0, 132.1 (d, *J* = 8.4 Hz), 131.9, 130.9, 128.9, 127.5 (d, *J* = 22.0 Hz), 127.2, 126.9, 123.0, 122.4, 118.3, 116.0, 115.8, 115.7, 46.2. HRMS (ESI) m/z calcd for C_25_H_16_^35^Cl_2_FNO_2_Na [M+Na]^+^ 474.0440, found 474.0443; C_25_H_16_^37^Cl_2_FNO_2_Na [M+Na]^+^ 476.0410, found 476.0406.

***3-benzoyl-4-(2,2-dichlorovinyl)-1-(2-methoxybenzyl)quinolin-2(1H)-one (3f)***

White solid after purification by column chromatography (petroleum ether/ethyl acetate = 15/1); 61.1 mg, 66% yield; mp: 116-118 ^o^C; ^1^H NMR (400 MHz, CDCl_3_) (δ, ppm): 7.99-7.95 (m, 2H), 7.76-7.74 (m, 1H), 7.66-7.61 (m, 1H), 7.59-7.55 (m, 1H), 7.53-7.49 (m, 2H), 7.41 (d, *J* = 8.4 Hz, 1H), 7.35-7.30 (m, 1H), 7.29-7.24 (m, 1H), 6.98-6.96 (m, 1H), 6.94-6.84 (m, 3H), 5.62 (s, 2H), 4.00 (s, 3H). ^13^C NMR (100 MHz, CDCl_3_) (δ, ppm): 193.7, 159.9, 156.7, 141.2, 139.7, 136.5, 133.7, 132.0, 131.3, 129.3, 128.6, 128.5, 127.2(8), 127.2(5), 127.1, 123.8, 122.8, 122.5, 121.0, 118.2, 115.8, 110.4, 55.6, 40.8. HRMS (ESI) m/z calcd for C_26_H_19_^35^Cl_2_NO_3_Na [M+Na]^+^ 486.0640, found 486.0635; C_26_H_19_^37^Cl_2_NO_3_Na [M+Na]^+^ 488.0610, found 488.0607.

***3-benzoyl-4-(2,2-dichlorovinyl)-1-(3-fluorobenzyl)quinolin-2(1H)-one (3g)***

White solid after purification by column chromatography (petroleum ether/ethyl acetate = 15/1); 46.9 mg, 52% yield; mp: 124-126 ^o^C; ^1^H NMR (300 MHz, CDCl_3_) (δ, ppm): 7.95 (d, *J* = 7.8 Hz, 2H), 7.76 (d, *J* = 8.1 Hz, 1H), 7.65-7.57 (m, 2H), 7.53-7.48 (m, 2H), 7.39-7.29 (m, 3H), 7.08 (d, *J* = 7.8 Hz, 1H), 7.00-6.95 (m, 2H), 6.88 (s, 1H), 5.58 (s, 2H). ^13^C NMR (100 MHz, CDCl_3_) (δ, ppm): 193.4, 163.2 (d, *J* = 247.0 Hz), 159.7, 141.6, 139.4, 138.4 (d, *J* = 7.1 Hz), 136.3, 133.8, 132.1, 131.2, 130.5 (d, *J* = 8.4 Hz), 129.2, 128.7, 127.5, 127.4, 123.1, 122.5 (d, *J* = 2.8 Hz), 122.3, 118.3, 115.4, 114.6 (d, *J* = 21.1 Hz), 113.9 (d, *J* = 22.3 Hz), 45.8. HRMS (ESI) m/z calcd for C_25_H_16_^35^Cl_2_FNO_2_Na [M+Na]^+^ 474.0440, found 474.0435; C_25_H_16_^37^Cl_2_FNO_2_Na [M+Na]^+^ 476.0410, found 476.0406.

***3-benzoyl-1-(3-chlorobenzyl)-4-(2,2-dichlorovinyl)quinolin-2(1H)-one (3h)***

White solid after purification by column chromatography (petroleum ether/ethyl acetate = 15/1); 53.2 mg, 57% yield; mp: 130-132 ^o^C; ^1^H NMR (300 MHz, CDCl_3_) (δ, ppm): 7.95 (d, *J* = 7.5 Hz, 2H), 7.76 (d, *J* = 7.8 Hz, 1H), 7.65-7.57 (m, 2H), 7.53-7.48 (m, 2H), 7.39-7.31 (m, 2H), 7.27 (d, *J* = 5.7 Hz, 3H), 7.19-7.16 (m, 1H), 6.89 (s, 1H), 5.55 (s, 2H). ^13^C NMR (100 MHz, CDCl_3_) (δ, ppm): 193.4, 159.8, 141.6, 139.4, 138.0, 136.3, 134.9, 133.8, 132.1, 131.2, 130.2, 129.2, 128.7, 127.9, 127.5, 127.4, 127.0, 125.1, 123.1, 122.3, 118.3, 115.3, 45.7. HRMS (ESI) m/z calcd for C_25_H_16_^35^Cl_3_NO_2_Na [M+Na]^+^ 490.0144, found 490.0141; C_25_H_16_^37^Cl_3_NO_2_Na [M+Na]^+^ 492.0115, found 492.0118.

***3-benzoyl-4-(2,2-dichlorovinyl)-1-(4-methylbenzyl)quinolin-2(1H)-one (3i)***

White solid after purification by column chromatography (petroleum ether/ethyl acetate = 15/1); 54.5 mg, 61% yield; mp: 150-152 ^o^C; ^1^H NMR (400 MHz, CDCl_3_) (δ, ppm): 8.00-7.93 (m, 2H), 7.76-7.74 (m, 1H), 7.67-7.56 (m, 2H), 7.55-7.46 (m, 3H), 7.35-7.30 (m, 1H), 7.22 (d, *J* = 8.0 Hz, 2H), 7.16 (d, *J* = 7.6 Hz, 2H), 6.89 (s, 1H), 5.57 (s, 2H), 2.35 (s, 3H). ^13^C NMR (100 MHz, CDCl_3_) (δ, ppm): 193.6, 159.8, 141.3, 139.6, 137.2, 136.4, 133.8, 132.9, 131.9, 131.3, 129.6, 129.3, 128.7, 127.3, 127.2, 126.9, 122.8, 122.5, 118.3, 115.6, 46.0, 21.1. HRMS (ESI) m/z calcd for C_26_H_19_^35^Cl_2_NO_2_Na [M+Na]^+^ 470.0691, found 470.0686; C_26_H_19_^37^Cl_2_NO_2_Na [M+Na]^+^ 472.0661, found 472.0662.

***3-benzoyl-4-(2,2-dichlorovinyl)-1-(4-fluorobenzyl)quinolin-2(1H)-one (3j)***

White solid after purification by column chromatography (petroleum ether/ethyl acetate = 15/1); 49.6 mg, 55% yield; mp: 128-130 ^o^C; ^1^H NMR (400 MHz, CDCl_3_) (δ, ppm): 7.95-7.91 (m, 2H), 7.74-7.72 (m, 1H), 7.63-7.56 (m, 2H), 7.50-7.46 (m, 2H), 7.41-7.39 (m, 1H), 7.34-7.27 (m, 2H), 7.25 (s, 1H), 7.03-6.99 (m, 2H), 6.85 (s, 1H), 5.53 (s, 2H). ^13^C NMR (100 MHz, CDCl_3_) (δ, ppm): 193.5, 162.2 (d, *J* = 246.3 Hz), 159.8, 141.4, 139.5, 136.3, 133.8, 132.0, 131.6 (d, *J* = 3.3 Hz), 131.3, 129.2, 128.8, 128.7(9), 128.7(8), 127.4 (d, *J* = 10.0 Hz), 123.0, 122.3, 118.3, 115.8 (d, *J* = 21.7 Hz), 115.4, 45.6. HRMS (ESI) m/z calcd for C_25_H_16_^35^Cl_2_FNO_2_Na [M+Na]^+^ 474.0440, found 474.0438; C_25_H_16_^37^Cl_2_FNO_2_Na [M+Na]^+^ 476.0410, found 476.0414.

***3-benzoyl-1-(4-chlorobenzyl)-4-(2,2-dichlorovinyl)quinolin-2(1H)-one (3k)***

White solid after purification by column chromatography (petroleum ether/ethyl acetate = 15/1); 49.5 mg, 53% yield; mp: 139-141 ^o^C; ^1^H NMR (300 MHz, CDCl_3_) (δ, ppm): 7.95 (d, *J* = 7.5 Hz, 2H), 7.76 (d, *J* = 7.8 Hz, 1H), 7.65-7.57 (m, 2H), 7.53-.7.48 (m, 2H), 7.39-7.28 (m, 3H), 7.27-7.13 (m, 3H), 6.89 (s, 1H), 5.55 (s, 2H). ^13^C NMR (100 MHz, CDCl_3_) (δ, ppm):193.4, 159.8, 141.5, 139.4, 136.3, 134.4, 133.9, 133.5, 132.0, 131.3, 129.2, 129.1, 128.7, 128.4, 127.5, 127.4, 123.1, 122.3, 118.3, 115.3, 45.6. HRMS (ESI) m/z calcd for C_25_H_16_^35^Cl_3_NO_2_Na [M+Na]^+^ 490.0144, found 490.0141; C_25_H_16_^37^Cl_3_NO_2_Na [M+Na]^+^ 492.0115, found 492.0118.

***3-benzoyl-1-(4-bromobenzyl)-4-(2,2-dichlorovinyl)quinolin-2(1H)-one (3l)***

White solid after purification by column chromatography (petroleum ether/ethyl acetate = 15/1); 49.0 mg, 48% yield; mp: 116-118 ^o^C; ^1^H NMR (300 MHz, CDCl_3_) (δ, ppm): 7.95 (d, *J* = 7.8 Hz, 2H), 7.76 (d, *J* = 8.1 Hz, 1H), 7.65-7.57 (m, 2H), 7.53-7.48 (m, 2H), 7.39-7.31 (m, 3H), 7.08 (d, *J* = 7.87 Hz, 1H), 7.01-6.94 (m, 2H), 6.88 (s, 1H), 5.58 (s, 2H). ^13^C NMR (100 MHz, CDCl_3_) (δ, ppm): 193.4, 159.8, 141.5, 139.4, 136.3, 134.9, 133.9, 132.0, 129.3, 129.2, 128.7, 128.1, 127.9, 127.5, 127.4, 123.1, 122.3, 121.5, 118.3, 115.3, 45.7. HRMS (ESI) m/z calcd for C_25_H_16_^35^Cl_2_^79^BrNO_2_Na [M+Na]^+^ 533.9639, found 533.9635; C_25_H_16_^37^Cl_2_^81^BrNO_2_Na [M+Na]^+^ 535.9619, found 535.9620.

***3-benzoyl-1-benzyl-4-(2,2-dichlorovinyl)-6-methylquinolin-2(1H)-one (3m)***

White solid after purification by column chromatography (petroleum ether/ethyl acetate = 15/1); 36.7 mg, 41% yield; mp: 152-154 ^o^C; ^1^H NMR (400 MHz, CDCl_3_) (δ, ppm): 8.00-7.95 (m, 2H), 7.66-7.61 (m, 1H), 7.53-7.49 (m, 3H), 7.42-7.31 (m, 5H), 7.29 (s, 2H), 6.88 (s, 1H), 5.59 (s, 2H), 2.46 (s, 3H). ^13^C NMR (100 MHz, CDCl_3_) (δ, ppm): 193.7, 159.7, 141.2, 137.6, 136.5, 136.0, 133.7, 133.3, 132.6, 129.3, 128.9, 128.6, 127.5, 127.0, 126.9(9), 126.9(6), 122.6, 118.3, 115.5, 46.1, 20.8. HRMS (ESI) m/z calcd for C_26_H_19_^35^Cl_2_NO_2_Na [M+Na]^+^ 470.0691, found 470.0690; C_26_H_19_^37^Cl_2_NO_2_Na [M+Na]^+^ 472.0661, found 472.0666.

***3-benzoyl-1-benzyl-4-(2,2-dichlorovinyl)-7-methylquinolin-2(1H)-one (3n)***

White solid after purification by column chromatography (petroleum ether/ethyl acetate = 15/1); 41.1 mg, 46% yield; mp: 143-145 ^o^C; ^1^H NMR (400 MHz, CDCl_3_) (δ, ppm): 7.98-7.95 (m, 2H), 7.66-7.61 (m, 2H), 7.53-7.49 (m, 2H), 7.39-7.29 (m, 6H), 7.16-7.14 (m, 1H), 6.90 (s, 1H), 5.59 (s, 2H), 2.47 (s, 3H). ^13^C NMR (100 MHz, CDCl_3_) (δ, ppm): 193.8, 160.0, 143.0, 141.4, 139.8, 136.6, 136.0, 133.7, 130.1, 129.2, 128.9, 128.6, 127.5, 127.2, 127.0, 126.9, 124.3, 122.7, 116.1, 115.7, 46.1, 22.2. HRMS (ESI) m/z calcd for C_26_H_19_^35^Cl_2_NO_2_Na [M+Na]^+^ 470.0691, found 470.0688; C_26_H_19_^37^Cl_2_NO_2_Na [M+Na]^+^ 472.0661, found 472.0668.

***3-benzoyl-4-(2,2-dichlorovinyl)-1-methylquinolin-2(1H)-one (3o)***

White solid after purification by column chromatography (petroleum ether/ethyl acetate = 15/1); 42.1 mg, 59% yield; mp: 128-130 ^o^C; ^1^H NMR (400 MHz, CDCl_3_) (δ, ppm): 7.98-7.92 (m, 2H), 7.78-7.71 (m, 2H), 7.66-7.60 (m, 1H), 7.54-7.47 (m, 3H), 7.42-7.36 (m, 1H), 6.86 (s, 1H), 3.80 (s, 3H). ^13^C NMR (100 MHz, CDCl_3_) (δ, ppm): 193.6, 159.6, 140.7, 140.1, 136.4, 133.8, 132.0, 131.4, 129.3, 128.7, 127.3, 122.8, 122.4, 118.0, 114.8, 29.6. HRMS (ESI) m/z calcd for C_19_H_13_^35^Cl_2_NO_2_Na [M+Na]^+^ 380.0221, found 380.0224; C_19_H_13_^37^Cl_2_NO_2_Na [M+Na]^+^ 382.0192, found 382.0196.

***3-benzoyl-1-benzyl-4-(2,2-dibromovinyl)quinolin-2(1H)-one (3p)***

White solid after purification by column chromatography (petroleum ether/ethyl acetate = 15/1); 67.8 mg, 65% yield; mp: 128-130 ^o^C; ^1^H NMR (400 MHz, CDCl_3_) (δ, ppm): 7.91-7.84 (m, 2H), 7.67-7.65 (m, 1H), 7.54-7.37 (m, 5H), 7.33 (d, *J* = 8.4 Hz, 1H), 7.23-7.21 (m, 3H), 7.20-7.14 (m, 3H), 5.49 (d, *J* = 73.2 Hz, 2H). ^13^C NMR (100 MHz, CDCl_3_) (δ, ppm): 193.5, 159.9, 143.7, 139.7, 136.4, 135.9, 133.8, 132.0, 131.2, 130.7, 129.4, 128.9, 128.6, 127.5, 127.5, 126.9, 122.9, 117.8, 115.6, 96.4, 46.2. HRMS (ESI) m/z calcd for C_25_H_17_^79^Br_2_NO_2_Na [M+Na]^+^ 543.9524, found 543.9520; C_25_H_17_^81^Br_2_NO_2_Na [M+Na]^+^ 545.9503, found 545.9510.

***1-benzyl-4-(2,2-dibromovinyl)-3-(4-methoxybenzoyl)quinolin-2(1H)-one (3q)***

White solid after purification by column chromatography (petroleum ether/ethyl acetate = 15/1); 60.7 mg, 55% yield; mp: 103-105 ^o^C; ^1^H NMR (400 MHz, CDCl_3_) (δ, ppm): 7.97 (d, *J* = 8.8 Hz, 1H), 7.78-7.76 (m, 1H), 7.59-7.55 (m, 1H), 7.50 (s, 1H), 7.44 (d, *J* = 8.4 Hz, 1H), 7.38-7.29 (m, 6H), 7.03-6.94 (m, 2H), 5.61 (d, *J* = 82.4 Hz, 2H), 3.91 (s, 3H). ^13^C NMR (100 MHz, CDCl_3_) (δ, ppm): 191.9, 164.2, 159.9, 143.3, 139.6, 135.9, 131.9, 131.8, 131.3, 131.0, 129.5, 128.9, 127.5, 127.4, 126.9, 122.8, 117.8, 115.6, 114.0, 96.2, 55.5, 46.2. HRMS (ESI) m/z calcd for C_26_H_19_^79^Br_2_NO_3_Na [M+Na]^+^ 573.9629, found 573.9626; C_26_H_19_^81^Br_2_NO_3_Na [M+Na]^+^ 575.9609, found 575.9616.

***1-benzyl-4-(2,2-dibromovinyl)-3-(4-fluorobenzoyl)quinolin-2(1H)-one (3r)***

White solid after purification by column chromatography (petroleum ether/ethyl acetate = 15/1); 51.8 mg, 48% yield; mp: 101-103 ^o^C; ^1^H NMR (400 MHz, CDCl_3_) (δ, ppm): 8.05-7.99 (m, 2H), 7.79-7.77 (m, 1H), 7.63-7.56 (m, 1H), 7.52 (s, 1H), 7.46 (d, *J* = 8.8 Hz, 1H), 7.40-7.30 (m, 6H), 7.21-7.17 (m, 2H), 5.61 (d, *J* = 85.6 Hz, 2H). ^13^C NMR (100 MHz, CDCl_3_) (δ, ppm): 192.0, 166.2 (d, *J* = 255.8 Hz), 159.8, 144.0, 139.7, 135.8, 132.1, 132.1, 132.0, 131.2, 130.3, 128.9, 127.6 (d, *J* = 4.2 Hz), 126.8, 123.0, 117.8, 115.9 (d, *J* = 22.2 Hz), 115.6, 100.0, 96.3, 46.2. HRMS (ESI) m/z calcd for C_25_H_16_^79^Br_2_FNO_2_Na [M+Na]^+^ 561.9430, found 561.9426; C_25_H_16_^81^Br_2_FNO_2_Na [M+Na]^+^ 563.9409, found 563.9411.

***3-benzoyl-4-(2,2-dibromovinyl)-1-methylquinolin-2(1H)-one (3s)***

White solid after purification by column chromatography (petroleum ether/ethyl acetate = 15/1); 53.5 mg, 60% yield; mp: 130-132 ^o^C; ^1^H NMR (400 MHz, CDCl_3_) (δ, ppm): 8.00-7.93 (m, 2H), 7.81-7.71 (m, 2H), 7.66-7.60 (m, 1H), 7.54-7.44 (m, 4H), 7.42-7.36 (m, 1H), 3.80 (s, 3H). ^13^C NMR (100 MHz, CDCl_3_) (δ, ppm): 193.6, 159.6, 143.1, 140.2, 136.4, 133.7, 132.0, 131.2, 129.4, 128.6, 127.5, 122.8, 117.6, 114.8, 96.4, 29.8. HRMS (ESI) m/z calcd for C_19_H_13_^79^Br_2_NO_2_Na [M+Na]^+^ 467.9211, found 467.9212; C_19_H_13_^81^Br_2_NO_2_Na [M+Na]^+^ 469.9190, found 469.9199.

***1-benzyl-3-(dibromomethyl)-4-(2,2-dichlorovinyl)quinolin-2(1H)-one (4a)***

White solid after purification by column chromatography (petroleum ether/ethyl acetate = 15/1); 54.0 mg, 54% yield; mp: 135-137 ^o^C; ^1^H NMR (300 MHz, CDCl_3_) (δ, ppm): 7.79 (d, *J* = 8.1 Hz, 1H), 7.64-7.41 (m, 2H), 7.37-7.25 (m, 8H), 5.79-5.46 (m, 2H). ^13^C NMR (100 MHz, CDCl_3_) (δ, ppm): 158.5, 139.2, 135.7, 132.3, 128.9, 127.6, 127.6, 126.7, 126.7, 123.1, 121.7, 115.5, 115.5, 47.0, 31.4. HRMS (ESI) m/z calcd for C_19_H_13_^79^Br_2_^35^Cl_2_NONa [M+Na]^+^ 523.8618, found 523.8619; C_19_H_13_^81^Br_2_^37^Cl_2_NONa [M+Na]^+^ 525.8589, found 525.8586.

***1-benzyl-3-(dibromomethyl)-4-(2,2-dichlorovinyl)-7-methylquinolin-2(1H)-one (4b)***

White solid after purification by column chromatography (petroleum ether/ethyl acetate = 15/1); 52.4 mg, 51% yield; mp: 156-158 ^o^C; ^1^H NMR (300 MHz, CDCl_3_) (δ, ppm): 8.02-7.62 (m, 2H), 7.43-7.24 (m, 6H), 7.18-7.12 (m, 2H), 5.78-5.48 (m, 2H), 2.45 (s, 3H). ^13^C NMR (100 MHz, CDCl_3_) (δ, ppm): 158.6, 143.4, 139.4, 135.8, 128.9, 127.5(9), 127.5(5), 126.7(0), 126.7(7), 124.6, 121.8, 115.6(9), 115.6(6), 46.9, 29.7, 22.2. HRMS (ESI) m/z calcd for C_20_H_15_^79^Br_2_^35^Cl_2_NONa [M+Na]^+^ 537.8775, found 537.8778; C_20_H_15_^81^Br_2_^37^Cl_2_NONa [M+Na]^+^ 539.8745, found 539.8748.

***1-benzyl-3-(dibromomethyl)-4-(2,2-dibromovinyl)-7-methylquinolin-2(1H)-one (4c)***

White solid after purification by column chromatography (petroleum ether/ethyl acetate = 15/1); 68.9 mg, 57% yield; mp: 153-155 ^o^C; ^1^H NMR (400 MHz, CDCl_3_) (δ, ppm): 7.74-7.44 (m, 2H), 7.40-7.35 (m, 2H), 7.32-7.27 (m, 4H), 7.18-7.11 (m, 2H), 5.77-5.47 (m, 2H), 2.44 (s, 3H). ^13^C NMR (100 MHz, CDCl_3_) (δ, ppm): 158.5, 143.4, 139.4, 135.8, 130.5, 128.9, 127.7, 127.5, 126.7, 124.6, 115.6, 97.4, 46.8, 31.5, 22.3. HRMS (ESI) m/z calcd for C_20_H_15_^79^Br_4_NONa [M+Na]^+^ 625.7764, found 625.7755; C_20_H_15_^81^Br_4_NONa [M+Na]^+^ 627.7744, found 627.7742.

**^1^H NMR Spectrum of Compound 3a (CDCl_3_)**

**^13^C NMR Spectrum of Compound 3a (CDCl_3_)**

**^1^H NMR Spectrum of Compound 3b (CDCl_3_)**

**^13^C NMR Spectrum of Compound 3b (CDCl_3_)**

**^1^H NMR Spectrum of Compound 3c (CDCl_3_)**

**^13^C NMR Spectrum of Compound 3c (CDCl_3_)**

**^1^H NMR Spectrum of Compound 3d (CDCl_3_)**

**^13^C NMR Spectrum of Compound 3d (CDCl_3_)**

**^1^H NMR Spectrum of Compound 3e (CDCl_3_)**

**^13^C NMR Spectrum of Compound 3e (CDCl_3_)**

**^1^H NMR Spectrum of Compound 3f (CDCl_3_)**

**^13^C NMR Spectrum of Compound 3f (CDCl_3_)**

**^1^H NMR Spectrum of Compound 3g (CDCl_3_)**

**^13^C NMR Spectrum of Compound 3g (CDCl_3_)**

**^1^H NMR Spectrum of Compound 3h (CDCl_3_)**

**^13^C NMR Spectrum of Compound 3h (CDCl_3_)**

**^1^H NMR Spectrum of Compound 3i (CDCl_3_)**

**^13^C NMR Spectrum of Compound 3i (CDCl_3_)**

**^1^H NMR Spectrum of Compound 3j (CDCl_3_)**

**^13^C NMR Spectrum of Compound 3j (CDCl_3_)**

**^1^H NMR Spectrum of Compound 3k (CDCl_3_)**

**^13^C NMR Spectrum of Compound 3k (CDCl_3_)**

**^1^H NMR Spectrum of Compound 3l (CDCl_3_)**

**^13^C NMR Spectrum of Compound 3l (CDCl_3_)**

**^1^H NMR Spectrum of Compound 3m (CDCl_3_)**

**^13^C NMR Spectrum of Compound 3m (CDCl_3_)**

**^1^H NMR Spectrum of Compound 3n (CDCl_3_)**

**^13^C NMR Spectrum of Compound 3n (CDCl_3_)**

**^1^H NMR Spectrum of Compound 3o (CDCl_3_)**

**^13^C NMR Spectrum of Compound 3o (CDCl_3_)**

**^1^H NMR Spectrum of Compound 3p (CDCl_3_)**

**^13^C NMR Spectrum of Compound 3p (CDCl_3_)**

**^1^H NMR Spectrum of Compound 3q (CDCl_3_)**

**^13^C NMR Spectrum of Compound 3q (CDCl_3_)**

**^1^H NMR Spectrum of Compound 3r (CDCl_3_)**

**^13^C NMR Spectrum of Compound 3r (CDCl_3_)**

**^1^H NMR Spectrum of Compound 3s (CDCl_3_)**

**^13^C NMR Spectrum of Compound 3s (CDCl_3_)**

**^1^H NMR Spectrum of Compound 4a (CDCl_3_)**

**^13^C NMR Spectrum of Compound 4a (CDCl_3_)**

**^1^H NMR Spectrum of Compound 4b (CDCl_3_)**

**^13^C NMR Spectrum of Compound 4b (CDCl_3_)**

**^1^H NMR Spectrum of Compound 4c (CDCl_3_)**

**^13^C NMR Spectrum of Compound 4c (CDCl_3_)**

**^1^H NMR Spectrum of Compound 5 (CDCl_3_)**

**^13^C NMR Spectrum of Compound 5 (CDCl_3_)**
